# Smooth Moves: Comparing Log Dimensionless Jerk Metrics from Body Center of Mass Trajectory and Wearable Sensor Acceleration During Walking

**DOI:** 10.3390/s25041233

**Published:** 2025-02-18

**Authors:** Paolo Brasiliano, Gaspare Pavei, Elena Bergamini

**Affiliations:** 1 Department of Motor, Human and Health Sciences, University of Rome “Foro Italico”, 00135 Roma, Italy; 2 Department of Pathophysiology and Transplantation, University of Milan, 20133 Milano, Italy; gaspare.pavei@unimi.it; 3 Department of Management, Information and Production Engineering, University of Bergamo, 24129 Bergamo, Italy; elena.bergamini@unibg.it

**Keywords:** movement smoothness, inertial measurement unit (IMU), body center of mass, gait analysis, LDLJ

## Abstract

Movement smoothness is a critical metric for evaluating motor control and sensorimotor impairments, with increasing relevance in neurorehabilitation and everyday functional assessments. This study investigates the correlation between two smoothness metrics (Log Dimensionless Jerk): LDLJV, derived from body center of mass (BCoM) trajectories using a gold-standard stereophotogrammetric system, and LDLJA, calculated from acceleration data recorded via an inertial measurement unit (IMU) placed at the L1–L2 level. Ten healthy adults (six men and four women; height: 1.71 ± 0.08 m; body mass: 68.2 ± 10.2 kg; age: 34.5 ± 8.5 years) walked on a treadmill at seven different speeds, with stride-specific data analyzed to compute smoothness indices for three anatomical components (antero-posterior, medio-lateral, cranio-caudal). Concordance between the metrics was evaluated using Bland–Altman analysis, Spearman’s correlation, and the mean absolute percentage error. The results revealed weak correlations and substantial biases across all components and speeds, reflecting inherent differences between IMU- and BCoM-derived data. Correcting biases improved alignment but did not eliminate discrepancies. The findings highlight that LDLJA captures only localized trunk accelerations, whereas BCoM-derived LDLJV approximates whole-body dynamics, making direct substitution infeasible. This study emphasizes the need for careful interpretation of IMU-based metrics and contributes to refining their application in real-world gait analyses.

## 1. Introduction

Movement smoothness plays an essential role in evaluating motor control and identifying sensorimotor impairments, providing valuable insights into movement quality [1,2]. Characterized by continuity and the absence of disruptions, smooth movements are recognized as indicators of healthy motor function and effective motor learning [3,4]. In the field of neurorehabilitation, smoothness has become a significant marker for assessing recovery, often showing strong correlations with functional improvements following neurological conditions such as stroke [1,5,6].

Traditionally, smoothness has been quantified in discrete and discontinuous movements—such as point-to-point reaching—using metrics based on jerk that consider kinematic profiles [7,8]. However, recent studies suggest the importance of extending smoothness analysis to cover a broader range of movements, including rhythmic and task-specific actions, which better reflect the complexity of everyday motor behavior [9,10].

This expanded approach has been recently adopted in clinical practice, where inertial measurement units (IMUs) have gained significant momentum due to their practicality and accessibility [11]. Compared to optoelectronic motion capture systems, which require controlled laboratory conditions, high-cost equipment, and expert operators, IMUs offer a cost-effective and accessible solution for motion analysis. One of the main advantages of IMUs is their ability to be used in ecological environments, allowing for gait assessments outside specialized facilities. Furthermore, IMUs do not require extensive setup time and can be deployed in routine clinical assessments with minimal infrastructure. Typically, a single IMU is placed at the lumbar level, leveraging its proximity to the body center of mass (BCoM) to approximate whole-body kinematics during gait. Research has shown that accelerations and displacements recorded at the lumbar level reflect BCoM behavior, offering an alternative to traditional, more complex systems like multi-segmental analyses or optical motion capture [12].

Motion smoothness has been evaluated using a large variety of metrics [6]. Among these, two metrics have gained widespread recognition for their reliability and robustness in quantifying smoothness: the Spectral Arc Length (SPARC) and the Log Dimensionless Jerk Velocity (LDLJV) [13,14]. The validity of these two metrics in measuring the smoothness of the BCoM trajectory has been assessed during gait at a self-selected speed in patients with stroke using an optoelectronic system [15]. In this context, the LDLJV has been proven more reliable [15].

Melendez-Calderon et al. [14] recently proposed a novel approach to address the limitations of using existing indices, such as the LDLJV, directly on IMU data, which can suffer from drift errors due to velocity reconstruction from accelerometer data [16,17,18]. To overcome this, they introduced a modified metric LDLJA designed to work with acceleration data, allowing for smoother estimation without the integration errors that often compromise translational movement analysis from IMUs. This new measure preserves critical smoothness properties, such as dimensionless scaling, and demonstrated robustness across simulated and experimental datasets. Consequently, LDLJA represents a valuable tool for assessing smoothness in IMU-based analyses, especially in cases where accurate velocity data are challenging to obtain. In addition to the findings derived from experimental data, the study by the authors demonstrated, using synthetic data, that while LDLJV and LDLJA yield different absolute values, these metrics are highly correlated, underscoring their consistency in reflecting smoothness characteristics under comparable conditions [14].

The objective of this study is to examine the hypothesis that the observed correlation remains consistent when analyzing LDLJV derived from the BCoM trajectory, as determined by a gold-standard stereophotogrammetric system, and LDLJA calculated from acceleration data acquired with an IMU placed at the lumbar level during human walking at a range of speeds. This analysis will help determine the extent to which LDLJA can serve as a reliable proxy for assessing movement smoothness in scenarios where direct BCoM velocity reconstruction is not feasible, while investigating the role of gait speed on the assessment itself. By addressing this question, this study seeks to further validate the robustness of LDLJA and its applicability in real-world contexts involving IMU-based analyses.

## 2. Materials and Methods

### 2.1. Participants and Experimental Setup

Ten healthy adults (six men and four women; height: 1.71 ± 0.08 m; body mass: 68.2 ± 10.2 kg; age: 34.5 ± 8.5 years) participated after providing informed consent, following the Declaration of Helsinki. This research was conducted under the approval of the University of Rome “Foro Italico” Ethics Committee (CAR 101/2021). Each participant walked on a treadmill (COSMED T170 DE MED, Rome, Italy) at seven distinct speeds (to address the whole range of walking speeds [19,20,21]): 0.28, 0.56, 0.83, 1.11, 1.39, 1.67, and 1.95 m/s (equivalent to 1 to 7 km/h, in 1 km/h increments), presented in a randomized sequence. Participants first stood still for five seconds before starting to walk at the designated speed. This initial phase of each trial was recorded to capture the static posture, which was necessary for the subsequent verticalization of the IMU data [22]. Dynamic data recording commenced once the treadmill achieved the required steady pace and lasted one minute. Treadmill accuracy was verified beforehand to ensure that the belt maintained the intended speed, which was monitored during the steady phase using a marker placed on one of the participants’ shoes.

### 2.2. Data Acquisition

A 6-camera stereophotogrammetric system (Vicon Vero, Oxford Metrics, UK) operating at 200 Hz recorded the 3D trajectories of 18 reflective markers attached at key joint centers, following the anatomical placements described by Minetti et al. and Pavei et al. [23,24,25]. Simultaneous measurements of the lower trunk’s 3D linear accelerations were obtained using a triaxial accelerometer (inertial measurement unit—IMU) (APDM Opal, Portland, OR, USA, 200 Hz, ±16 g), secured at the L1–L2 level. To ensure precise data synchronization between the systems, an electronic trigger box was used. The placement of both the markers and the accelerometer was carried out by the same skilled operator; markers were applied directly to the skin with double-sided tape, and the accelerometer was fastened with an elastic belt. Careful attention was paid to the fixation of the IMU on the trunk to limit its oscillations relative to the skeleton [26]. Moreover, the IMU was mounted so that its axes were parallel to the trunk anatomical axes.

### 2.3. Stereophotogrammetric Data Processing

The marker trajectories were processed with a zero-lag, second-order Butterworth low-pass filter, using a cut-off frequency determined through residual analysis for each marker’s coordinate [27]. To estimate the 3D trajectory of the BCoM, an 11-segment model based on Dempster’s inertial parameters for body segments was applied [24,28]. Left and right foot strike events were identified based on marker positions, following the method described by O’Connor et al. [29]. These events provided a basis for segmenting strides, which were subsequently used to compute smoothness indices both from optoelectronic and IMU data. The BCoM 3D velocity was obtained through the first derivative of the BCoM 3D position. Afterwards, BCoM 3D velocity signals were filtered using a zero-lag, fourth-order Butterworth low-pass filter with a cut-off frequency of 10 Hz and used to estimate the BCoM smoothness as follows [13]:

(1) $${LDLJV}_{BCoM}=-\ln\left(\frac{{\left(t_{2}-t_{1}\right)}^{3}}{v_{\mathrm{peak}}^{2}}\int_{t_{1}}^{t_{2}}\left.|\frac{d^{2}}{dt^{2}}v\left(t\right)\right.{|}^{2}\;dt\right)$$

where *v*(*t*) represents the BCoM velocity, *t* denotes time, *t*_1_ and *t*_2_ are the start and end instants of each stride, and *v*_peak_ is the peak velocity over each stride.

### 2.4. Inertial Sensor Data Processing

To ensure consistent initial conditions across all participants and establish a repeatable IMU-based reference system, a verticalized local reference frame was employed. During the initial static phase, a rigid transformation was calculated to align one axis of the IMU’s local frame with the gravity vector [22]. This transformation, which remained constant over time, was subsequently applied to the acceleration components recorded at each sampling instant during the movement. This procedure allowed us to express accelerometer data in a reference frame that approximated the anatomical antero-posterior (AP), medio-lateral (ML), and cranio-caudal (CC) axes [22]. A zero-lag, fourth-order Butterworth low-pass filter was used to process accelerometer data with a cut-off frequency of 10 Hz. Following this, the IMU-based smoothness was calculated as follows:

(2) $${LDLJA}_{IMU}\;=\;-\ln\left(\frac{t_{2}-t_{1}}{a_{\mathrm{peak}}^{2}}\int_{t_{1}}^{t_{2}}\left.|\frac{d}{dt}a\left(t\right)\right.{|}^{2}\;dt\right)$$

where *a* is the linear acceleration data and *t*_1_ and *t*_2_ are the starting and ending instants of each stride, and *a*_peak_ is the peak acceleration over each stride.

### 2.5. Statistics

The data analysis described in this section was carried out over all the identified gait strides within each walking speed. First, an outlier analysis was conducted for each index value, using a z-score threshold of ±3 to identify and remove outliers from the dataset. The data distribution for the three components of each index was tested using the Shapiro–Wilk test. Given the non-normal distribution of most of the data, the median and the interquartile range were used to summarize the data and provide information about their dispersion. Afterwards, the concordance between LDLJV_BCoM_ and LDLJA_IMU_ was assessed via Bland and Altman analysis [30] and using the mean absolute percentage error (MAPE) and Spearman’s correlation coefficient. The alpha level of significance was set at 0.05 for all statistical analyses.

## 3. Results

**Figure 1:**
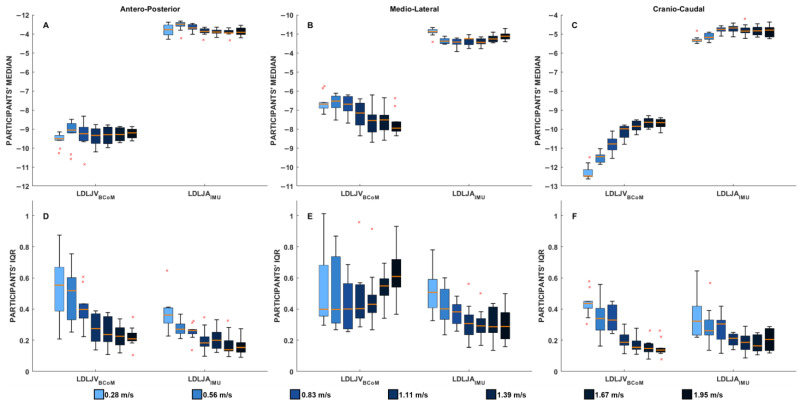
Box plots illustrating the median values (panels (**A**–**C**)) and interquartile range (IQR; panels (**D**–**F**)) of smoothness indices across walking speeds and spatial components. The smoothness indices include LDLJV_BCoM_, representing the smoothness of the center of mass derived using an optoelectronic system, and LDLJV_IMU_, representing the smoothness calculated from an IMU positioned at the L1–L2 vertebral level. The color gradient reflects increasing walking speeds, from lighter to darker shades. Red crosses represent outliers. Each column corresponds to a specific spatial component: antero-posterior, medio-lateral, and cranio-caudal.

A total of 3383 strides were analyzed, with the slowest walking speed (0.28 m/s) having 208 strides and the fastest (1.95 m/s) 627 strides. The median and interquartile range values of LDLJV_BCoM_ and LDLJA_IMU_ at each walking speed for the three components are shown in Figure 1.

There were consistent differences between the absolute values of LDLJA_IMU_ and LDLJV_BCoM_ across all anatomical axes, reflecting the inherent differences in both data source and processing between the two acquisition systems. These differences stem from the fact that LDLJA_IMU_ is derived from trunk accelerations measured by an IMU, whereas LDLJV_BCoM_ is based on the whole-body center of mass trajectory obtained via stereophotogrammetry. Furthermore, the two indices are computed from different underlying signals—acceleration for LDLJA_IMU_ and velocity for LDLJV_BCoM_—and, despite following similar principles, are formulated using slightly different equations (Equation (1) for LDLJV_BCoM_ and Equation (2) for LDLJA_IMU_). As a result, they capture distinct aspects of movement smoothness, leading to systematic discrepancies in their absolute values. Despite these absolute differences, most trends across speed were preserved.

In the AP component (Panel A), both LDLJA_IMU_ and LDLJV_BCoM_ exhibited relatively stable median values across walking speeds. This suggests that movement smoothness in the forward direction remains consistent regardless of gait speed, at least when assessed using these two different methods. However, LDLJA_IMU_ systematically yielded lower absolute values compared to LDLJV_BCoM_, which likely reflects the fundamental difference between acceleration-based and velocity-based smoothness computations. The IQR values (Panel D) for both indices showed a decreasing trend with increasing speed, indicating reduced stride-to-stride variability at higher speeds. This pattern suggests that walking at faster speeds promotes a more repeatable and stable movement pattern in the forward direction.

For the ML component (Panel B), substantial discrepancies between the two metrics were observed. LDLJV_BCoM_ exhibited a clear trend of decreasing median values with increasing walking speed, indicating a progressive reduction in smoothness in the medio-lateral direction. This may reflect the increasing challenge of stabilizing lateral movements at higher speeds. In contrast, LDLJA_IMU_ did not display a consistent pattern across speeds, with its median values remaining more stable across speeds. This discrepancy suggests that the IMU, placed at the lower trunk, may not be accurately capturing medio-lateral CoM dynamics, potentially due to localized trunk accelerations that do not fully represent whole-body movement. The IQR analysis (Panel E) further emphasizes this inconsistency: while LDLJV_BCoM_ showed an increasing variability trend with speed (indicating more pronounced differences between strides), LDLJA_IMU_ exhibited the opposite pattern, with IQR values decreasing at higher speeds. This suggests that the IMU-based metric may underestimate variability in lateral smoothness and fail to capture its true relationship with walking speed.

In the CC component (Panel C), both LDLJA_IMU_ and LDLJV_BCoM_ followed a similar trend, with increasing median values as speed increased. This alignment indicates that movement smoothness in the vertical direction improves at higher speeds. The IQR values (Panel F) also exhibited a decreasing pattern for both indices, reinforcing the idea that variability in cranio-caudal movement smoothness diminishes at higher speeds.

**Figure 2:**
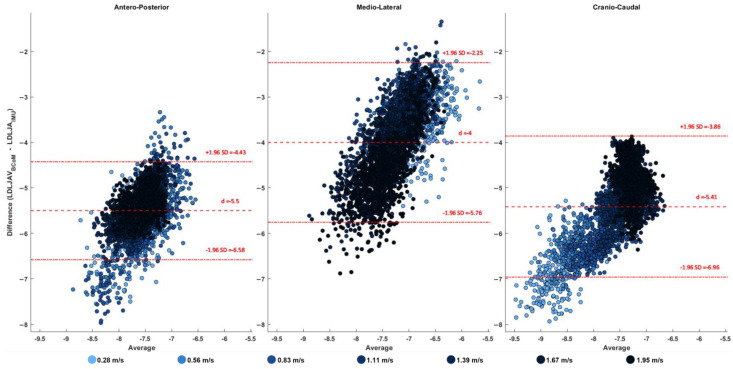
Bland–Altman plots illustrating the agreement between LDLJV_BCoM_ (smoothness derived from the body center of mass using an optoelectronic system) and LDLJV_IMU_ (smoothness derived from an IMU positioned at the L1–L2 vertebral level) for each spatial component: antero-posterior, medio-lateral, and cranio-caudal. The color gradient represents increasing walking speeds, from lighter to darker shades. Thick red dashed lines indicate the upper and lower limits of agreement (LoAs), while the thin red dashed line represents the mean bias (d).

The Bland–Altman analysis, represented in Figure 2, provided a deeper understanding of the relationship between the two metrics across all speeds. The bias and limits of agreement (LoAs; upper and lower) were calculated as averages across all walking speeds. Detailed speed-specific results are available in the Supplementary Materials.

For the AP component, the bias was calculated as d = −5.52, with upper and lower limits of agreement of −4.44 and −6.60, respectively. The distribution of the data points suggests a consistent agreement between the two systems across speeds, with no evident proportional bias. However, at lower speeds, a slight heteroscedasticity is observed, with greater variability in the differences. This heteroscedasticity decreases progressively as walking speed increases, with darker points still showing a positive trend, although more tightly clustered.

For the ML component, the overall bias was d = −4.03, with limits of agreement of −2.27 and −5.78. The scatter plot highlights a slightly heteroscedastic distribution of data points, with greater differences at lower values of the mean (i.e., for strides with less smooth movements). This indicates that the disagreement between the two systems tends to increase as smoothness decreases.

The CC component displayed a bias of d = −5.43, with lower and upper limits of agreement of −3.87 and −6.98, respectively. Unlike the other components, the distribution of the data points across speeds appeared similar, with no significant change in variability. However, the bias was influenced by walking speed: lighter blue points, representing slower speeds, were concentrated lower on the plot (indicating larger bias values), while darker blue points, representing faster speeds, were concentrated higher (indicating smaller bias values). The clustering of darker points near the bias line is not reflective of better agreement but rather of the higher number of strides recorded at faster speeds, which dominates the data distribution.

Table 1 shows the detailed results of the Bland–Altman analysis for all the walking speeds together with the Spearman’s correlation coefficient and MAPE. The MAPE metric was also calculated after removing the bias obtained from the Bland–Altman analysis (reported within brackets).

The Spearman’s correlation coefficient (ρ) values calculated for the AP, ML, and CC components across all walking speeds were consistently low, indicating poor agreement between LDLJA_IMU_ and LDLJV_BCoM_ in all cases. The highest Spearman’s r value was observed for the AP component (0.48) at 1.39 m/s, demonstrating the strongest, albeit still weak, correlation. None of the ML or CC component values exceeded this threshold, further underscoring the lack of strong linear association between the two metrics.

The MAPE revealed substantial discrepancies between the measurements, with values generally exceeding 50% across all components and speeds. However, correcting for the bias identified through the Bland–Altman analysis markedly improved the MAPE values. For example, the AP component showed a reduction in MAPE from 54.77% to 10.86% at 0.28 m/s, highlighting the significant impact of bias removal. Similar improvements were observed for the ML and CC components, with the lowest adjusted MAPE values recorded at 1.67 m/s for the AP component (6.17%) and at 0.28 m/s for the CC component (8.6%), reflecting better alignment after accounting for systematic differences.

The bias (d) values across speeds and components followed a relatively decreasing pattern for the AP and CC components. Vice versa, bias in the ML components increased with increasing speed. More specifically, the bias in the AP component ranged from −5.80 at 0.28 m/s to −5.36 at 1.94 m/s, indicating a modest reduction as speed increased. Conversely, the ML component exhibited a greater range, with the bias becoming more negative at higher velocities, transitioning from −3.62 at 0.28 m/s to −4.62 at 1.94 m/s. This trend suggests an increasing disagreement in the medio-lateral direction as walking speed rises. The CC component showed an overall reduction in the magnitude of bias, with values decreasing from −6.93 at 0.28 m/s to −4.84 at 1.94 m/s.

The upper and lower limits of agreement (LoAs) demonstrated a narrowing trend for the AP component as velocity increased, with their range shrinking from 2.54 at 0.28 m/s to 1.21 at 1.94 m/s. This indicates reduced variability and improved agreement between LDLJA_IMU_ and LDLJV_BCoM_ at higher speeds. In contrast, the ML and CC components exhibited wider bounds, with no clear narrowing trend (ML component) or a less pronounced one (CC component) across speeds.

## 4. Discussion

This study investigated the agreement between two smoothness metrics, LDLJA_IMU_ and LDLJV_BCoM_, during walking at seven different speeds. The two metrics quantify movement smoothness, relying on different data sources and processing methods. Specifically, LDLJA_IMU_ is based on the acceleration measured by an IMU located on the lower trunk, whereas LDLJV_BCoM_ relies on the trajectory, and then velocity, of the BCoM as obtained by the marker trajectory measured by a motion capture system. Through the combination of Bland–Altman, MAPE, and Spearman’s correlation analyses, the relationship between LDLJA_IMU_ and LDLJV_BCoM_ was quantified and analyzed for three anatomical components, i.e., AP, ML, and CC, to clarify if the former can reasonably approximate the latter. The results showed that there was no significant correlation between LDLJA_IMU_ and LDLJV_BCoM_ across components or speeds. Even the strongest correlation, observed in the AP component at 1.39 m/s (r = 0.48), was weak. This finding contrasts with the work of Melendez-Calderon et al. [14], who reported a strong correlation between these metrics using synthetic data. Our data suggest that the relationship between LDLJA_IMU_ and LDLJV_BCoM_ might be more context-dependent than previously thought, with differences emerging in more complex scenarios like real-world gait data. This discrepancy is not only influenced by the complexity of the context but also by the fundamental differences in what each metric measures. LDLJV_BCoM_ is derived from the velocity of the center of mass, capturing the overall dynamics of the body, whereas LDLJA_IMU_ is based on the acceleration of the lower trunk, reflecting more localized motion. This highlights that the relationship between these smoothness measures is inherently dependent not only on the context but also on the biomechanical variables being analyzed.

Beyond the lack of correlation, the two metrics were found to consistently differ in their absolute values. This aligns with Melendez-Calderon et al. [14], who emphasized that LDLJA_IMU_ and LDLJV_BCoM_ measure different aspects of smoothness. The high MAPE values observed in this study reinforce this distinction. However, removing the bias identified through the Bland–Altman analysis significantly improved the alignment between the two metrics. In general, after bias removal, MAPE values dropped from values ranging from 40 to 50% to values ranging from 10 to 20%, underscoring the importance of accounting for systematic differences when interpreting these measures. Nonetheless, the identified bias does not appear to be systematic, making it challenging to leverage this information for practical real-world applications.

The Bland–Altman analysis provided further insight into the nature of these discrepancies. The AP and CC components showed relatively stable biases across speeds, with the limits of agreement narrowing at higher velocities, suggesting that LDLJA_IMU_ performs better in these conditions. However, the ML component exhibited larger and more variable biases, particularly at higher speeds, indicating persistent challenges in measuring medio-lateral smoothness with a single IMU. These results suggest that while LDLJA_IMU_ may capture some aspects of smoothness, it cannot fully replicate LDLJV_BCoM_, particularly for movements in the ML direction. Moreover, heteroscedasticity suggests that the IMU-based measurement system might be less reliable for detecting subtle differences in smoothness, particularly for strides with lower smoothness values and at slower walking speeds.

The reported results must be read taking into consideration the following limitations. First, the analysis was conducted exclusively on healthy individuals, which limits the generalizability of the findings to clinical applications. Second, the study focused solely on the LDLJ metric, as LDLJA is the only smoothness index currently validated for IMU data. Third, the analysis was restricted to treadmill walking, making it challenging to generalize the results to overground gait. Finally, while the sample size was relatively small, the choice of treadmill walking allowed for the collection of a large number of gait cycles, enhancing the robustness of stride-level analyses.

Overall, our findings do not support the initial hypothesis that LDLJA_IMU_ could be used as an alternative to LDLJV_BCoM_. While the results of Melendez-Calderon et al. [14] demonstrated a correlation between these metrics, our study highlights that this relationship does not hold when focusing on the smoothness of the BCoM using a single IMU located at the lumbar level during walking. This supports previous studies reporting that a single sensor is not able to capture the complex dynamics of BCoM movement during walking [19,23].

## 5. Conclusions

This study evaluated the agreement between LDLJA and LDLJV and revealed that LDLJA cannot fully replicate LDLJV due to inherent differences between trunk accelerations and whole-body dynamics, particularly in the medio-lateral direction. These results provide valuable insights into the limitations of single-sensor approaches for approximating whole-body smoothness. By highlighting these discrepancies, this work advances the understanding of movement smoothness metrics and underscores the importance of considering data sources and processing methods in gait analysis. While these findings highlight the limitations of relying exclusively on IMU-based smoothness metrics as a direct substitute for BCoM-derived measures, they also point to potential—albeit still speculative—applications in clinical and everyday contexts. Should further research confirm their reliability, wearable sensors could help clinicians and caregivers detect subtle shifts in gait quality in individuals recovering from neurological conditions or in older adults at risk of mobility decline, allowing for timely intervention. In parallel, the feasibility of continuous remote monitoring could support more personalized rehabilitation plans and promote safer independent living. Nonetheless, it is important to acknowledge that the modest concordance observed in this study warrants caution. Broader validation is necessary to determine whether sensor-derived metrics can consistently inform clinical decision-making and sustain long-term mobility assessment beyond controlled laboratory settings.

## Figures and Tables

**Table 1:** Table report the Spearman’s correlation coefficient, the mean absolute percentage error (MAPE) before and after bis removal (the latter data is presented between parentheses), and the results of the Bland-Altman analysis for all the assessed walking speed.

	Spearman’s ρ			MAPE (MAPE: LDLJA-d vs. LDLJV)			BIAS			Upper LoA			Lower LoA		
	AP	ML	CC	AP	ML	CC	AP	ML	CC	AP	ML	CC	AP	ML	CC
0.28 m/s	0.05	0.09	0.18	54.77% (10.86%)	42.22% (12.46%)	56.62% (8.6%)	−5.80	−3.62	−6.93	−4.53	−2.22	−5.85	−7.07	−5.02	−8.01
0.56 m/s	0.20	0.15	0.31	55.22% (13.88%)	37.67% (8.87%)	55.52% (5.75%)	−5.68	−3.26	−6.38	−4.10	−2.09	−5.65	−7.27	−4.44	−7.11
0.83 m/s	−0.13	0.20	0.26	54.48% (13%)	37.72% (8.91%)	55.64% (7.75%)	−5.64	−3.33	−6.02	−4.12	−2.14	−5.13	−7.16	−4.52	−6.90
1.11 m/s	0.23	−0.09	0.27	53.15% (8.21%)	41.64% (12.2%)	53.27% (6.08%)	−5.54	−3.87	−5.40	−4.61	−2.37	−4.67	−6.46	−5.36	−6.14
1.39 m/s	0.48	−0.07	0.18	52.63% (7.64%)	43.24% (12.92%)	51.24% (6.43%)	−5.45	−4.18	−5.05	−4.60	−2.56	−4.29	−6.29	−5.81	−5.81
1.67 m/s	0.33	−0.05	−0.22	52.19% (6.17%)	44.88% (12.38%)	49.96% (6.84%)	−5.37	−4.34	−4.83	−4.68	−2.79	−4.04	−6.07	−5.89	−5.62
1.95 m/s	0.39	0.07	0.30	52.32% (5.1%)	47.10% (13.88%)	49.82% (7.44%)	−5.36	−4.62	−4.84	−4.75	−3.00	−3.93	−5.96	−6.25	−5.75

## Data Availability

The data presented in this study are available on request from the corresponding author due to privacy reasons.

## Supplementary Materials

The following supporting information can be downloaded at: https://www.mdpi.com/article/10.3390/s25041233/s1. The Bland-Altman plot of each walking speeds with the relative agreement analysis results are presented in supplementary material, together with the representation of the marker set used and the scheme of the experimental set up and procedure.
