# Supplementary material for: Smooth Moves: Comparing Log Dimensionless Jerk Metrics from Body Center of Mass Trajectory and Wearable Sensor Acceleration During Walking
Source: Sensors (Basel). 2025 Feb 18;25(4):1233. doi: 10.3390/s25041233 (PMC11860695; doi:10.3390/s25041233)
Supplement: Supplementary file 1 [file sensors-25-01233-s001.zip › sensors-3444737-supplementary.pdf]

WALKIN SPEED 0.28 m/s

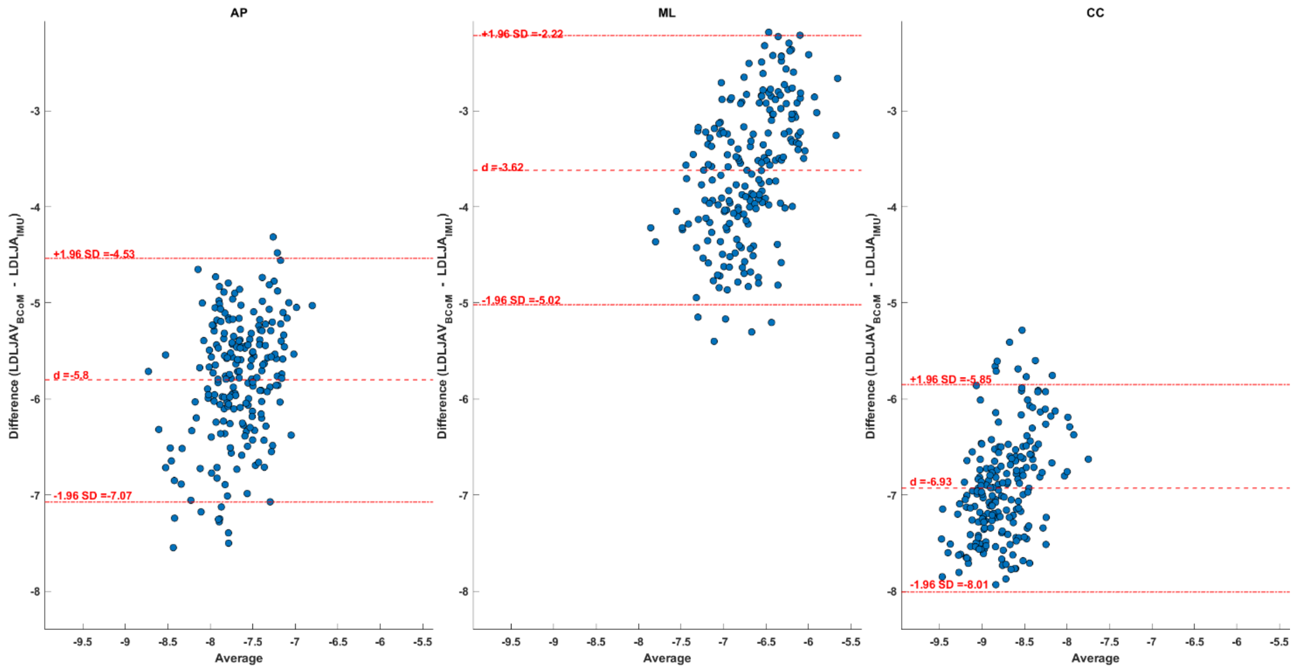

	Spearman's ϱ			MAPE<br>*(MAPE: LDLJA-d vs LDLJV)*			BIAS			Upper LoA			Lower LoA		
	AP	*ML*	*CC*	*AP*	*ML*	*CC*	*AP*	*ML*	*CC*	*AP*	*ML*	*CC*	*AP*	*ML*	*CC*
**0.28 m/s**	0,05	0,09	0,18	54,77%<br>*(10,86 %)*	42,22%<br>*(12,46%)*	56,62%<br>*(8,6%)*	-5,80	-3,62	-6,93	-4,53	-2,22	-5,85	-7,07	-5,02	-8,01

WALKING SPEED 0.56 m/s

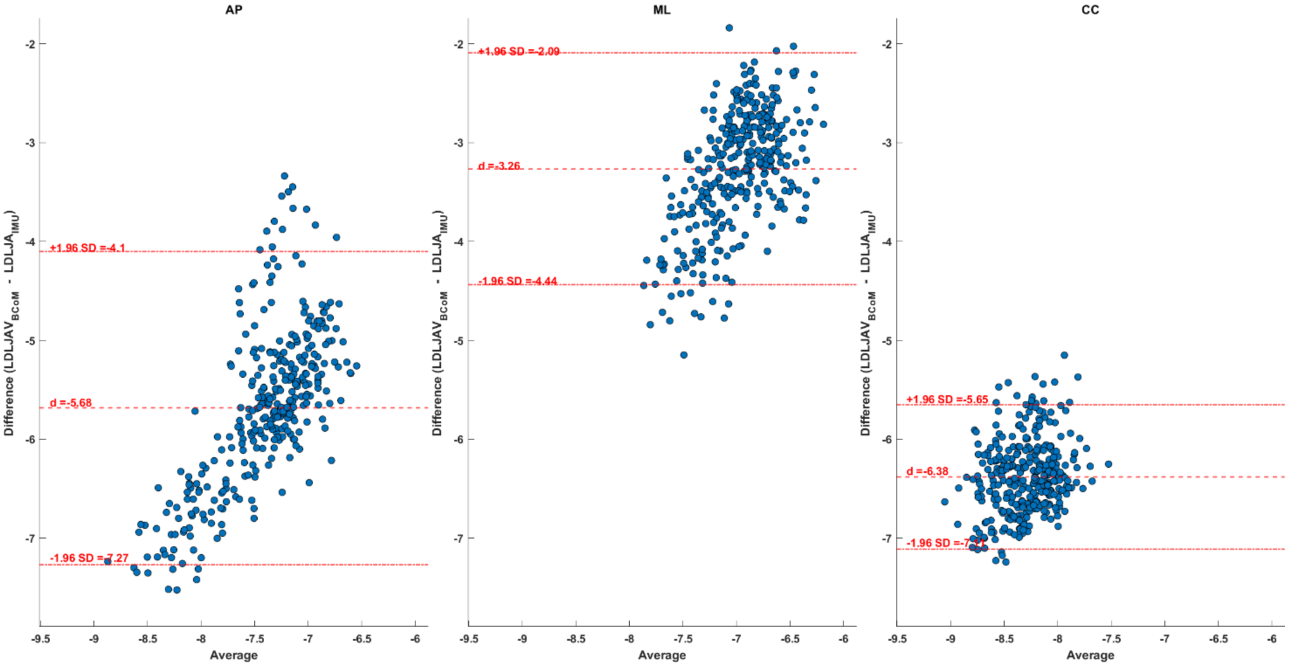

	**Spearman's ϱ**			**MAPE** *(MAPE: LDLJA-d vs LDLJV)*			**BIAS**			**Upper LoA**			**Lower LoA**		
	AP	*ML*	*CC*	*AP*	*ML*	*CC*	*AP*	*ML*	*CC*	*AP*	*ML*	*CC*	*AP*	*ML*	*CC*
**0.56 m/s**	0,20	0,15	0,31	55,22% *(13,88%)*	37,67% *(8,87%)*	55,52% *(5,75%)*	-5,68	-3,26	-6,38	-4,10	-2,09	-5,65	-7,27	-4,44	-7,11

WALKING SPEED 0.83 m/s

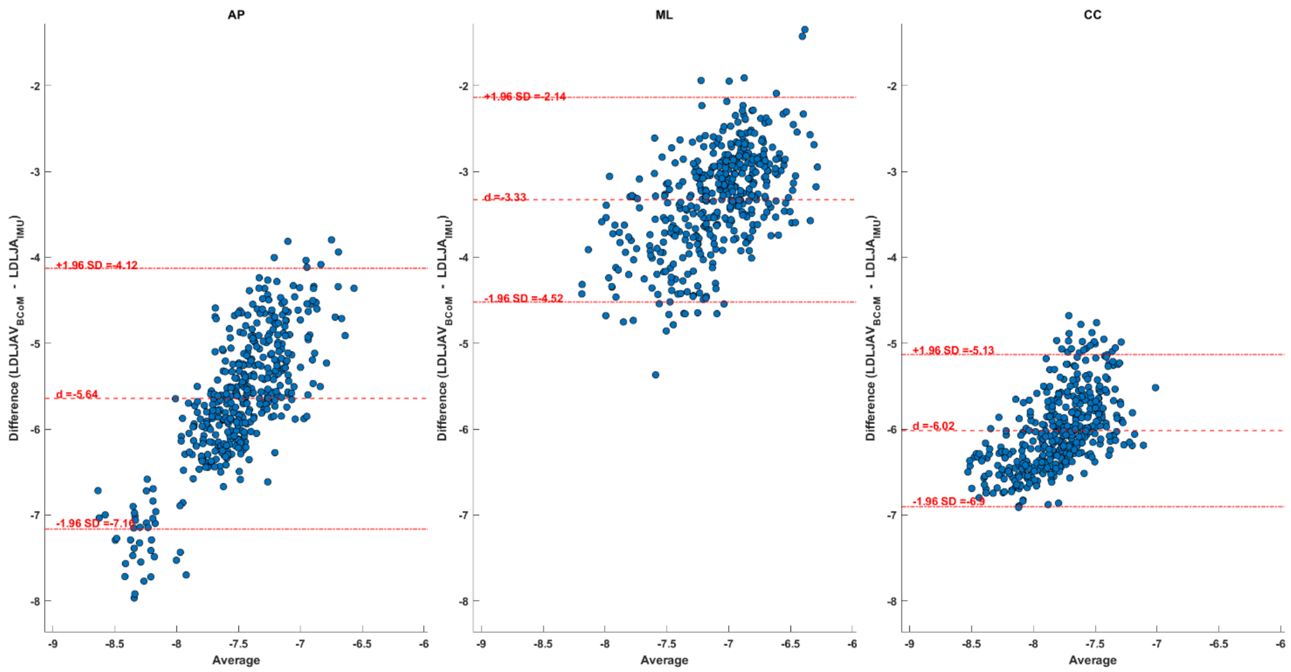

	Spearman's ϱ			MAPE (*MAPE: LDLJA-d vs LDLJV)*			BIAS			Upper LoA			Lower LoA		
	AP	*ML*	*CC*	*AP*	*ML*	*CC*	*AP*	*ML*	*CC*	*AP*	*ML*	*CC*	*AP*	*ML*	*CC*
**0.83 m/s**	-0,13	0,20	0,26	54,48% *(13%)*	37,72% *(8,91%)*	55,64% *(7,75%)*	-5,64	-3,33	-6,02	-4,12	-2,14	-5,13	-7,16	-4,52	-6,90

WALKING SPEED 1.11 m/s

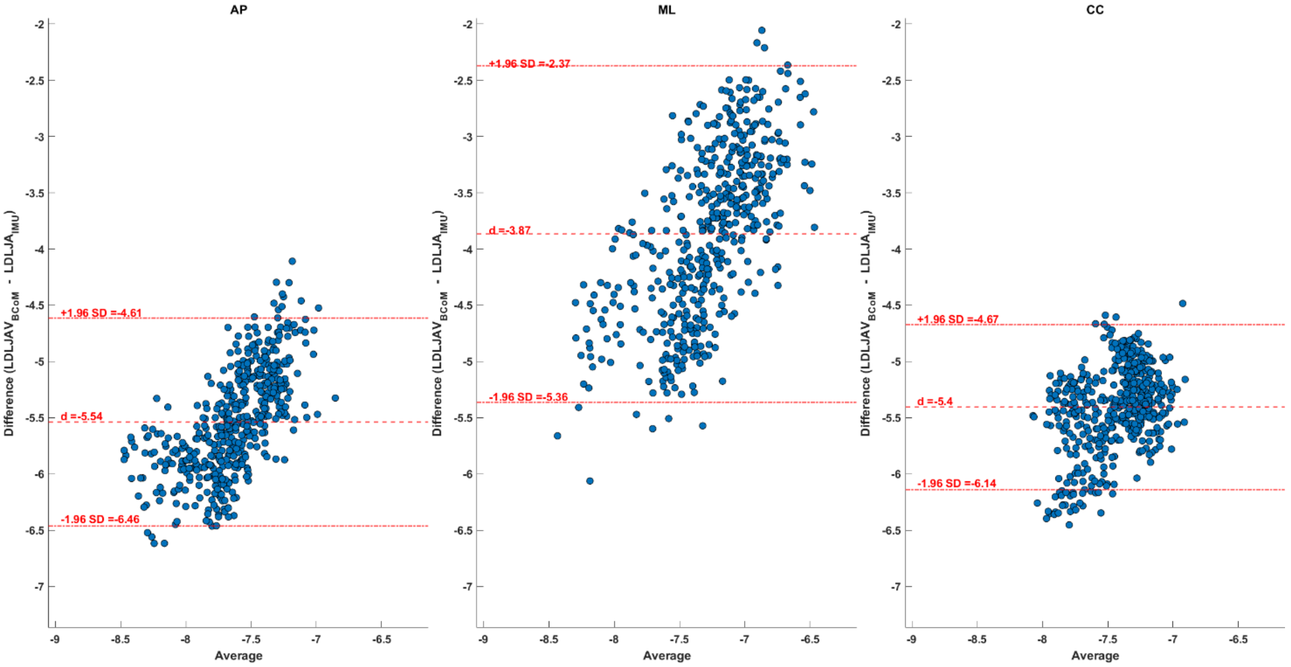

	Spearman's ϱ			MAPE<br>*(MAPE: LDLJA-d vs LDLJV)*			BIAS			Upper LoA			Lower LoA		
	AP	*ML*	*CC*	*AP*	*ML*	*CC*	*AP*	*ML*	*CC*	*AP*	*ML*	*CC*	*AP*	*ML*	*CC*
**1.11 m/s**	0,31	-0,03	0,30	53,15%<br>*(8,21%)*	41,64%<br>*(12,2%)*	53,27%<br>*(6.08%)*	-5,54	-3,87	-5,40	-4,61	-2,37	-4,67	-6,46	-5,36	-6,14

WALKING SPEED 1.39 m/s

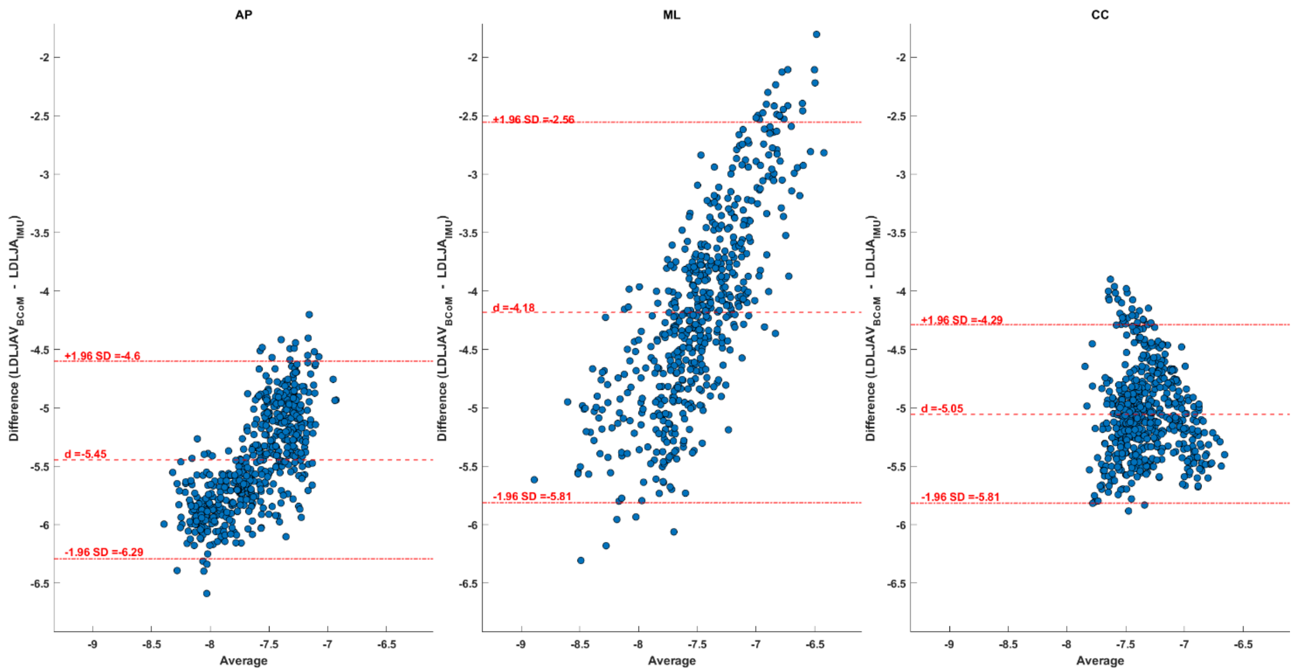

	Spearman's ϱ			MAPE<br>*(MAPE: LDLJA-d vs LDLJV)*			BIAS			Upper LoA			Lower LoA		
	AP	*ML*	*CC*	*AP*	*ML*	*CC*	*AP*	*ML*	*CC*	*AP*	*ML*	*CC*	*AP*	*ML*	*CC*
**1.39 m/s**	0,48	-0,07	0,18	52,63%<br>*(7,64%)*	43,24%<br>*(12,92%)*	51,24%<br>*(6,43%)*	-5,45	-4,18	-5,05	-4,60	-2,56	-4,29	-6,29	-5,81	-5,81

WALKING SPEED 1.67 m/s

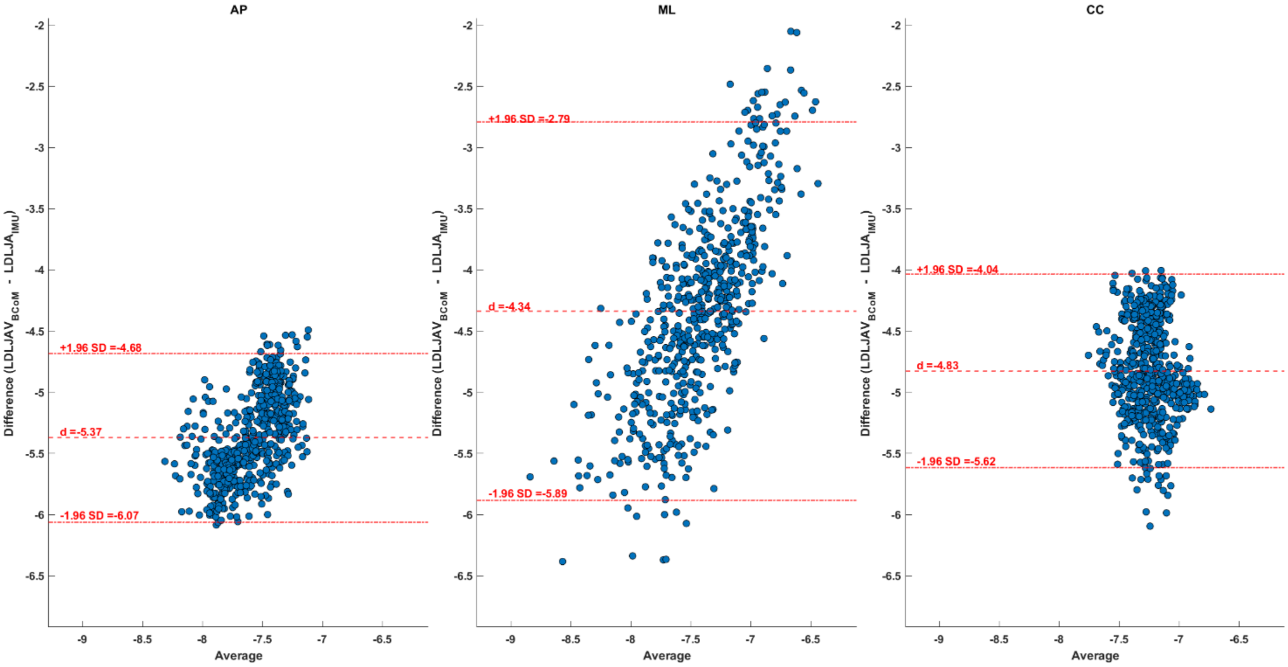

	**Spearman's ϱ**			**MAPE** *(MAPE: LDLJA-d vs LDLJV)*			**BIAS**			**Upper LoA**			**Lower LoA**		
	AP	*ML*	*CC*	*AP*	*ML*	*CC*	*AP*	*ML*	*CC*	*AP*	*ML*	*CC*	*AP*	*ML*	*CC*
**1.67 m/s**	0,36	-0,05	-0,21	52,19% *(6,17%)*	44,88% *(12,38%)*	49,96% *(6,84%)*	-5,37	-4,34	-4,83	-4,68	-2,79	-4,04	-6,07	-5,89	-5,62

WALKING SPEED 1.95 m/s

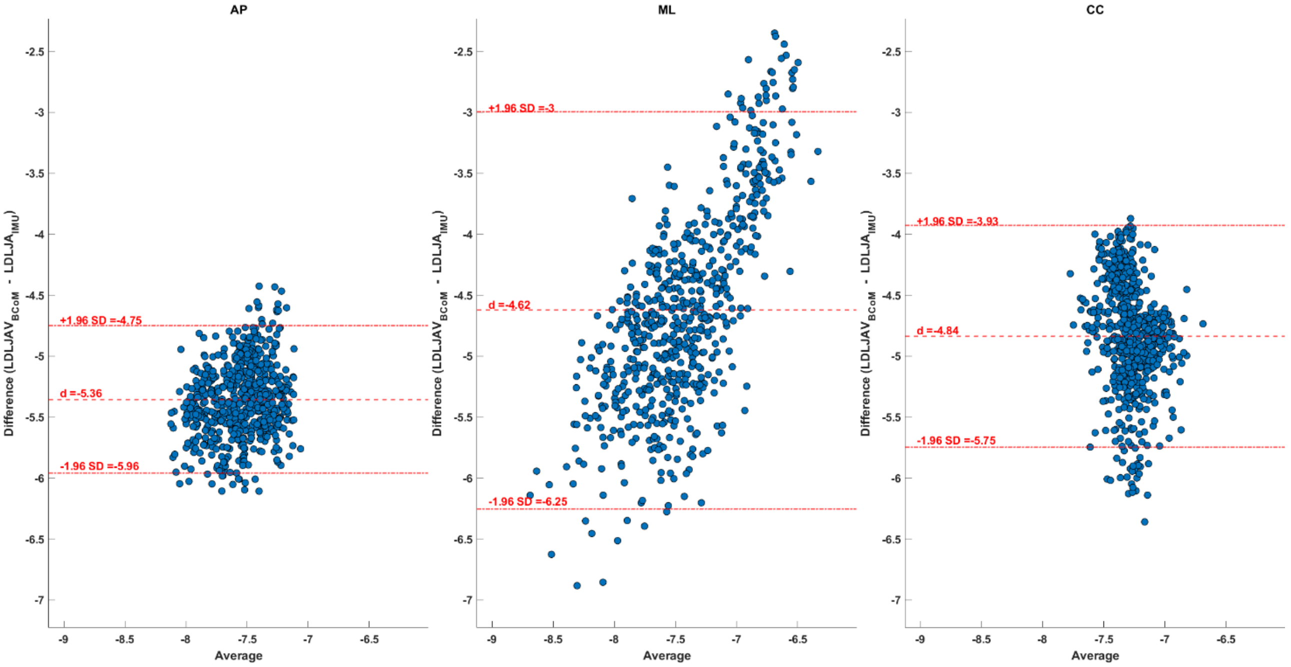

	**Spearman's ϱ**			**MAPE** *(MAPE: LDLJA-d vs LDLJV)*			**BIAS**			**Upper LoA**			**Lower LoA**		
	AP	*ML*	*CC*	*AP*	*ML*	*CC*	*AP*	*ML*	*CC*	*AP*	*ML*	*CC*	*AP*	*ML*	*CC*
**1.95 m/s**	0,39	0,07	0,30	52,32% *(5,1%)*	47,10% *(13,88%)*	49,82% *(7,44%)*	-5,36	-4,62	-4,84	-4,75	-3,00	-3,93	-5,96	-6,25	-5,75

EXPERIMENTAL SET UP AND PROCEDURE

Participant set up

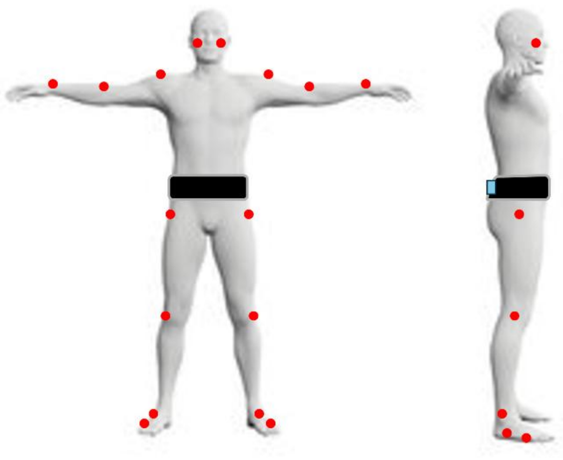

After signing the informed consent, participants were equipped with the markers and the IMU sensor as shown in the figure above.

Laboratory set up

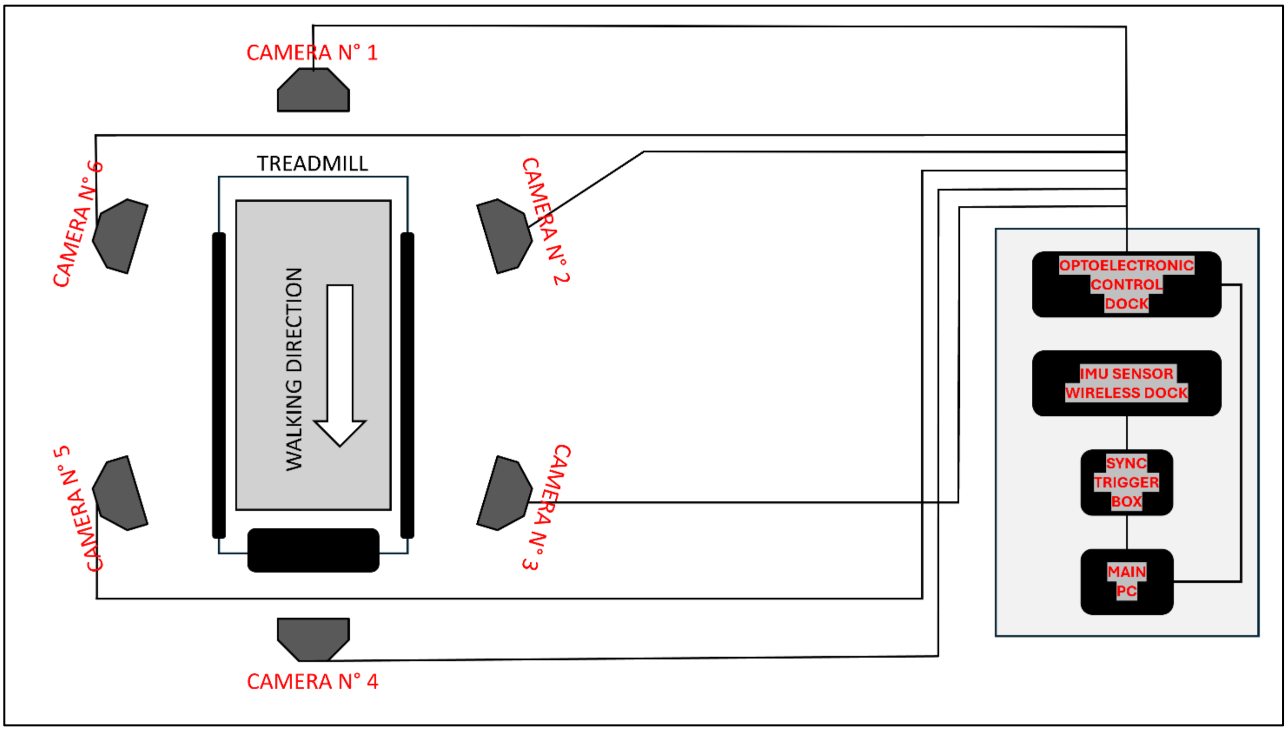

Figure above shows the laboratory set up

DATA ACQUISITION FLOWCHART

After optoelectronic system calibration and participant preparation, a random order of the seven selected walking speed was generated.

Afterwards, a familiarization trial was performed to get the participant comfortable while walking on the treadmill with the equipped marker set and IMU sensor.

At the beginning of each trial a static phase was recorded to verticalize IMU data recorded during the respective walking trial.

The participant started walking and walking speed was gradually increased until the desired speed was reached. Once the desired walking speed was reached the recording was started from the main PC (see laboratory set up figure). Each trial lasted one minute. At the end walking speed was gradually decreased until the treadmill stopped.

Between trials, the participants were allowed to rest as needed before starting the following trial.
